# Glycemic variability and glucose complexity in critically ill patients: a retrospective analysis of continuous glucose monitoring data

**DOI:** 10.1186/cc11657

**Published:** 2012-10-02

**Authors:** Richard Brunner, Gabriel Adelsmayr, Harald Herkner, Christian Madl, Ulrike Holzinger

**Affiliations:** 1 Department of Medicine III, Division of Gastroenterology and Hepatology, ICU 13H1, Medical University of Vienna, Waehringer Guertel 18-20, 1090 Vienna, Austria; 2 Department of Emergency Medicine, Medical University of Vienna, Waehringer Guertel 18-20, 1090 Vienna, Austria

## Abstract

**Introduction:**

Glycemic variability as a marker of endogenous and exogenous factors, and glucose complexity as a marker of endogenous glucose regulation are independent predictors of mortality in critically ill patients. We evaluated the impact of real time continuous glucose monitoring (CGM) on glycemic variability in critically ill patients on intensive insulin therapy (IIT), and investigated glucose complexity - calculated using detrended fluctuation analysis (DFA) - in ICU survivors and non-survivors.

**Methods:**

Retrospective analysis were conducted of two prospective, randomized, controlled trials in which 174 critically ill patients either received IIT according to a real-time CGM system (n = 63) or according to an algorithm (n = 111) guided by selective arterial blood glucose measurements with simultaneously blinded CGM for 72 hours. Standard deviation, glucose lability index and mean daily delta glucose as markers of glycemic variability, as well as glucose complexity and mean glucose were calculated.

**Results:**

Glycemic variability measures were comparable between the real time CGM group (n = 63) and the controls (n = 111). Glucose complexity was significantly lower (higher DFA) in ICU non-survivors (n = 36) compared to survivors (n = 138) (DFA: 1.61 (1.46 to 1.68) versus 1.52 (1.44 to 1.58); *P *= 0.003). Diabetes mellitus was significantly associated with a loss of complexity (diabetic (n = 33) versus non-diabetic patients (n = 141) (DFA: 1.58 (1.48 to 1.65) versus 1.53 (1.44 to 1.59); *P *= 0.01).

**Conclusions:**

IIT guided by real time CGM did not result in significantly reduced glycemic variability. Loss of glucose complexity was significantly associated with mortality and with the presence of diabetes mellitus.

## Introduction

Glucose control in critically ill patients has been a highly disputed topic since 2001, when van den Berghe *et al*. showed that intensive insulin therapy (IIT) (mean glucose levels ≤6.11 mmol/L) could reduce the morbidity and mortality of patients in surgical ICUs by 42% [1]. However, subsequent studies came to inconclusive findings [2]. Recently, several retrospective trials found glycemic variability *per se *to be associated with mortality in critically ill patients, independent of mean glucose concentration [3-9].

The measure glycemic variability describes fluctuations of blood glucose over time. As glucose fluctuations are not covered by mean glucose, glycemic variability has been suggested as an additional measure for glucose control. Glycemic variability is represented by standard deviation (SD), mean daily δ blood glucose or glucose lability index (GLI). SD is the most commonly used parameter and is calculated as the square-root of the average of the squared differences between individual glucose values and the mean. Mean daily δ blood glucose describes the mean of the daily difference between minimum and maximum blood glucose. These two measures do not take order and timing of measurements into account. GLI is the squared difference between consecutive blood glucose levels per unit of actual time between the samples. GLI considers the time between and the order of measurements. Although no gold standard of measuring glycemic variability has been established yet, SD seems to be the best predictor of mortality [10].

In a prospective study of septic critically ill patients a significant association between high glycemic variability and mortality was found [11]. These results are consistent with *in vitro *data showing that short-time fluctuations of glucose levels induce endothelial cell damage and apoptosis [12]. Moreover, a significant association between glycemic variability and 8-iso prostaglandin F_2α_, a marker of oxidative stress and potential mediator of organ dysfunction, has been shown in diabetic type 2 patients [13]. Minimal glycemic variability has been proposed to become the gold standard of glycemic control in diabetic patients [14].

Glycemic variability depends on both endogenous patient-specific factors such as severity of disease and diabetes status [15], as well as exogenous factors such as type and quality of glucose monitoring, the glucose algorithm used for the calculation of the insulin rate, compliance of the nursing staff with the recommendations of the protocol and application of medication including enteral and parenteral nutrition. As the endogenous glucose regulation system can hardly be influenced, glycemic variability needs to be improved by acting on the exogenous factors. Besides well-trained nursing staff and continuous application of medication, appropriate glucose monitoring is suggested to minimize blood glycemic variability. Therefore, the need for real time continuous glucose level reporting has been emphasized numerous times [16,17]. Subcutaneous continuous glucose monitoring (CGM) systems provide both real time capability and adequate accuracy in medical critically ill patients including those requiring vasopressors [18].

Although IIT may be associated with increased glycemic variability [7] we hypothesized that IIT guided by real time glucose monitoring would decrease glycemic variability. Thus, we aimed to evaluate the impact of real time CGM on glycemic variability in critically ill patients.

More importantly, by using CGM devices valuable insights into glucose regulation are possible. While glucose variability can be calculated using conventional blood glucose measurements every four to six hours, glucose complexity calculation requires the availability of continuous glucose data. Recently, in critically ill patients glucose complexity has been proposed as a marker of endogenous glucose regulation [19].

Glucose complexity is a dynamic measure of glucose time series and, therefore, seems to provide more powerful information on endogenous glucose regulation than does conventional glycemic variability analysis. In contrast to glycemic variability that describes the magnitude of glucose fluctuations over several hours, glucose complexity is proposed as a measure of short-term glucose oscillations. Glycemic variability depends on endogenous and exogenous factors, whereas glucose complexity is proposed as a description of the endogenous glucose regulation system that is independent from exogenous factors. Lundelin *et al*. hypothesized that in a healthy regulatory system, glucose levels are corrected frequently and result in a 'complex' glucose profile [19]. However, in critically ill patients a decomplexification of glucose regulation was suggested [20]. Low complexity represents the inability of the patient to correct glucose fluctuations frequently and quickly.

In a small number of patients glycemic profile was shown to be more complex in ICU survivors than in ICU non-survivors [19]. However, this has not been confirmed in a large group of critically ill patients. Therefore, we investigated the role of glucose complexity in a large group of critically ill patients.

The primary hypothesis is that real-time CGM guidance of IIT is associated with decreased glycemic variability in critically ill patients. The secondary hypothesis of the study is that glucose complexity is independently associated with increased mortality.

## Materials and methods

Data analysis was approved by the ethics committee of the Medical University of Vienna. Because of the retrospective character of the analysis the need for informed consent was waived by the institutional review board.

### Patients and setting

This is a *post-hoc *analysis of two prospective, randomized controlled trials conducted in an eight-bed closed medical ICU at the University Hospital of Vienna, Austria [21,22]. During the period from April 2005 to August 2008 a total of 983 critically ill patients were admitted, 728 of whom were ventilated. A total of 174 consecutive, mechanically ventilated and sedated patients fulfilling the inclusion criteria (age ≥18, expected to stay ≥48 hours in the ICU after initiation of IIT) were enrolled in the study within 48 hours after ICU admission. Patients were not enrolled in the study if any of the following criteria were present: ICU stay expected to be <48 hours, mechanical ventilation not expected for >48 hours, inclusion in another study, no CGM device available during the screening phase or glucose values in the normal range without insulin therapy.

The original objectives of the two studies were to evaluate the impact of circulatory shock requiring norepinephrine therapy on the accuracy and reliability of a subcutaneous CGM sensor in critically ill patients [21] and to evaluate the impact of real-time CGM on glycemic control and risk of hypoglycemia in critically ill patients [22].

### Research design

To evaluate the impact of real time CGM on glucose variability, data on patients allocated to two groups (real time CGM, n = 63 and controls, n = 111) were analyzed.

All included patients were treated with IIT to maintain glucose levels between 4.44 and 6.11 mmol/L.

In the control group insulin infusion rates were guided by selective arterial blood glucose measurements, obtained using an automated blood gas analyzer (Radiometer ABL 700®, Copenhagen, Denmark). IIT was performed by the nursing staff according to a previously described dynamic paper-based insulin titration algorithm [23] based on the algorithm used in the Leuven studies [1,24]. This algorithm prescribes the insulin infusion rate, time of next glucose measurement (between one and six hours), and, in the case of hypoglycemia, dextrose administration depending on glucose levels and glucose trends. Consequently, it defines nine different states requiring different actions, although leaving space for interpretation by the responsible nurse [22]. In the control group glucose levels were additionally recorded continuously using the Continuous Glucose Monitoring System® (CGMS, Medtronic MiniMed, Northridge, CA, USA), but were blinded and available only in retrospect.

In the real time CGM group, IIT was performed by the nursing staff and insulin infusion rates were guided by continuously available glucose levels using the Guardian® real time CGMS (Medtronic MiniMed) according to the algorithm used in the control group. In contrast to the control group, nurses were requested to take real time glucose readings in close intervals according to clinical necessity at personal discretion, however at least every two hours [22].

To investigate glucose complexity detrended fluctuation analysis (DFA) [19] was calculated for ICU survivors (n = 138) and ICU non-survivors (n = 36). Furthermore, we evaluated glucose complexity in diabetic (n = 33) and non-diabetic (n = 141) patients.

### Real time continuous glucose monitoring system (real time CGMS)

The Guardian® real time CGMS has been described in detail previously [22]. Briefly, it displays a mean of 30 glucose measurements over the last five minutes on a monitor, allowing glucose monitoring in real time. The real time CGMS was calibrated against blood glucose measurements, obtained using an automated blood gas analyzer, at least four times per day (every five to six hours). Sensors were planned to stay in place for 72 hours.

### Continuous glucose monitoring system (CGMS)

The CGMS has been described in detail previously [22]. Briefly, it is equivalent to the Guardian® real time CGMS, except it is lacking the capability to display glucose concentration in real time. Glucose concentrations were recorded continuously in an internal monitor blinded to the study team and obtained after the trial for further analysis.

### Statistical analysis

To evaluate the impact of real time CGM on glucose variability as a marker of exogenous glucose regulation, we used linear regression analyses with glycemic variability, represented as SD (Glu_SD_), glucose lability index ({∑(Glu_n _- Glu_n+1 _(mmol/L))^2^*(h_n+1 _- h_n_)^-1^)*(number of readings)^-1 ^or mean daily delta (difference between minimum and maximum) glucose as primary outcome and real time versus concealed CGMS (controls) as predictor.

Secondary endpoints were coefficient of variation (CV) of glucose (Glu_SD_/Glucose mean (%)), variability or mean of glucose during the first 24 hours, maximum glucose during ICU stay.

To investigate glycemic dynamics and its relation with mortality in critically ill patients we used glucose complexity as the main risk factor and ICU mortality as outcome. Glucose complexity is proposed as the representation of the endogenous glucoregulatory process. Although it is similar to glucose variability it is able to detect minor systemic alterations in endogenous glucose regulation. In a healthy regulatory system, glucose levels are corrected frequently and result in a 'complex' glucose profile. However, in critically ill patients a decomplexification of glucose regulation was suggested [20].

Glucose complexity was calculated using DFA. DFA is a unitless metric that estimates the degree of long-range correlations within a signal, analyzing how the time series and its linear regression diverge as the 'time window' considered increases. As a rule of thumb, higher complexities are displayed as lower DFA (until a minimum of 0.5). Details on DFA can be found elsewhere [19].

Glucose complexity was normally distributed as assessed by visual inspection of a histogram but not linearly related with mortality as assessed with a test for deviation from linearity in a logistic regression model. We found a good fit with a quadratic function and entered a quadratic term into the model as a consequence. We assessed whether this association was modified by diabetes by testing the significance of the interaction term in such a model using a Wald test. To allow for the influence of other variables on the effect of glucose complexity we entered SAPS II (Simplified Acute Physiology Score) score, age (years), sex, and diabetes (yes versus no) as covariables into our model. These covariables were selected *a priori*. As we merged patients from two studies into this database we used a multivariate mixed effects logistic regression model to additionally allow for potential clustering within each of the two studies. As sensitivity analyses we used robust estimations instead which yielded virtually the same results.

We assessed the impact of the method of glucose determination (CGMS versus blood gas analyzer (BGA)) on the glucose variability measures SD, CV, GLI and mean daily delta glucose by calculating them from CGMS and BGA values in all patients.

Data are presented as mean ± standard deviation, median (25^th ^to 75^th ^percentile) or absolute count and relative frequency. For bivariate comparisons we tabulated data and used simple one-way analysis of variance (ANOVA), Mann-Whitney rank sum test or a chi2-test as appropriate to test the null hypothesis of no difference.

For data management and analyses we used Excel for Mac 2011 and STATA 11.0 for Mac (Stata Corp., College Station, TX, USA). Generally a two-sided *P*-value <0.05 was considered statistically significant.

## Results

### Baseline characteristics

Baseline characteristics of 174 critically ill patients receiving IIT either guided by a real time CGM system or by selective arterial blood glucose measurements with simultaneously blinded CGM can be found in Table 1. Mean CGM time was 59.2 ± 14.7 hours (RT CGM group); 7.0 ± 1.6 BGA readings per patient in 24 hours were taken in the control group.

**Table 1:** Admission reason and baseline characteristics.

	**Real time CGM **[21]	**Controls **[21,22]	**Total **[21,22]
Included Patients	63	111	174
Admission reason	Number of patients (% of patients in the category)		
Respiratory failure	15 (24)	23 (21)	38 (22)
CPR	12 (19)	27 (24)	39 (22)
Sepsis/Septic shock	13 (20)	24 (22)	37 (21)
Heart failure	8 (13)	21 (19)	29 (17)
Neurologic disease/Coma	9 (14)	10 (9)	19 (11)
Pulmonary embolism	3 (5)	3 (3)	6 (3)
GI-bleeding/ALF	3 (5)	2 (2)	5 (3)
Necrotising pancreatitis	0 (0)	1 (1)	1 (1)
History of diabetes	12 (19)	21 (19)	33 (19)
			
Age (years)	59 ± 15	62 ± 16	61 ± 16
Gender (male/female)	41/22	64/47	105/69
BMI (kg/m²)	27.1 ± 5.1	26.4 ± 3.9	26.7 ± 4.4
SAPS II	60 ± 16	58 ± 16	59 ± 16
SOFA on admission day	11.5 ± 3.8	10.9 ± 3.5	11 ± 4

Data are means ± SD, unless otherwise stated. ALF, acute liver failure; BMI, body mass index; CPR, Cardiopulmonary resuscitation; SAPSII, Simplified Acute Physiology Score; SD, standard deviation; SOFA, Sequential Organ Failure Assessment.

### Differences in glycemic metrics between patients using real time CGM and controls

The use of real time CGM did not have any impact on the measures of glycemic variability, glucose complexity and maximum glucose (Table 2).

**Table 2:** Glycemic metrics in the real time CGM and control group.

	Real time CGM **(number = 63) **[21]	Controls **(number = 111) **[21,22]	*P*-value
Measures of glycemic variability			
Variability of glucose (SD) (mmol/L)	1.19 ± 0.49	1.27 ± 0.54	0.330
Variability of glucose (GLI)	81 (43 to 197)	126 (64 to 222)	0.247
Variability of glucose (δ) (mmol/L)	4.47 ± 2.02	4.76 ± 2.07	0.336
Coefficient of variation (%)	20 ± 7	21 ± 8	0.547
Variability of glucose during first 24 hours (SD) (mmol/L)	0.84 (0.65 to 1.33)	1.04 (0.66 to 1.40)	0.395
Variability of glucose during first 24 hours (GLI)	85 (38 to 190)	118 (60 to 207)	0.348
Variability of glucose during first 24 hours (δ) (mmol/L)	5.72 ± 2.42	5.73 ± 2.40	0.966
Measures of glucose			
Mean of glucose during first 24 hours (mmol/L)	5.70 (5.19 to 6.47)	5.96 (5.48 to 6.36)	0.099
Maximum glucose (mmol/L)	9.43 ± 2.12	9.77 ± 2.26	0.329
Glucose complexity	1.54 ± 0.11	1.52 ± 0.11	0.210
Measures of IIT			
Cumulative daily dose of insulin (I.U.)	45 ± 27	40 ± 23	0.239
Number of changes of the insulin infusion/24 hours	3.8 ± 1.5	3.6 ± 1.3	0.573
Extent of insulin change (%)	57 ± 19	62 ± 23	0.150
Number of BGA measurements/24 hours	7.4 ± 2.1	7.0 ± 1.6	0.217
Number of BGA not required by the algorithm/24 h^a^	0 (0 to 0)	0 (0 to 1)	0.238

^a^safety/double check BGA measurements. Data are shown as mean ± SD or median (25^th ^to 75^th ^percentile). BGA, blood gas analyzer; CGM, continuous glucose monitoring; GLI, glucose lability index; IIT, intensive insulin therapy.

### Differences in glycemic metrics between ICU survivors and non-survivors

Measures of glycemic variability, mean glucose and hypoglycemia were similar between ICU survivors and ICU non-survivors, whereas glucose complexity was significantly lower in non-survivors (Table 3).

**Table 3:** Glycemic metrics in ICU survivors and non-survivors.

	**Real Time CGM and controls **[21,22]		
	**Survivors (number = 138)**	**Non-survivors (number = 36)**	***P*-value**

Measures of glycemic variability			
Variability of glucose (SD) (mmol/L)	1.21 ± 0.49	1.39 ± 0.59	0.067
Variability of glucose (GLI)	112 (62 to 214)	126 (56 to 223)	0.468
Variability of glucose (δ) (mmol/L)	4.54 ± 2.00	5.08 ± 2.20	0.158
Coefficient of variation (%)	20 ± 7	22 ± 8	0.169
Variability of glucose during first 24 hours (SD) (mmol/L)	0.92 (0.64 to 1.32)	1.03 (0.73 to 1.46)	0.083
Variability of glucose during first 24 hours (GLI)	101 (54 to 200)	123 (54 to 198)	0.232
Variability of glucose during first 24 hours (δ) (mmol/L)	5.55 ± 2.23	6.42 ± 2.91	0.051
Measures of glucose			
Mean of glucose (mmol/L)	6.03 ± 0.57	6.23 ± 0.09	0.097
Mean of glucose during first 24 hours (mmol/L)	5.89 (5.40 to 6.36)	5.74 (5.27 to 6.39)	0.414
Maximum glucose (mmol/L)	9.52 ± 2.01	10.16 ± 2.82	0.121
Glucose complexity	1.51 ± 0.10	1.58 ± 0.14	0.003
Measures of hypoglycemia			
Time below 4.44 mmol/L (min/24hours)	109 (27 to 262)	120 (29 to240)	0.874
Time below 3.33 mmol/L (min/24hours)	0 (0 to 29)	0 (0 to 50)	0.864
Time below 2.22 mmol/L (min/24 hours)	0 (0 to 0)	0 (0 to 0)	0.116

Data are shown as mean ± SD or median (25^th ^to 75^th ^percentile). CGM, continuous glucose monitoring; GLI, glucose lability index; SD, standard deviation.

These measures were similar in hospital survivors and non-survivors (data not shown).

### Diabetic status and glucose complexity

The presence of diabetes was significantly associated with a loss of complexity (higher DFA) (diabetic (n = 33) versus non-diabetic patients (n = 141): DFA 1.58 (1.48 to 1.65) versus 1.53 (1.44 to 1.59); *P *= 0.01). This difference persisted even after correcting for survival (*P *= 0.027).

### Multivariate analysis of glucose complexity and mortality

**Figure 1:**
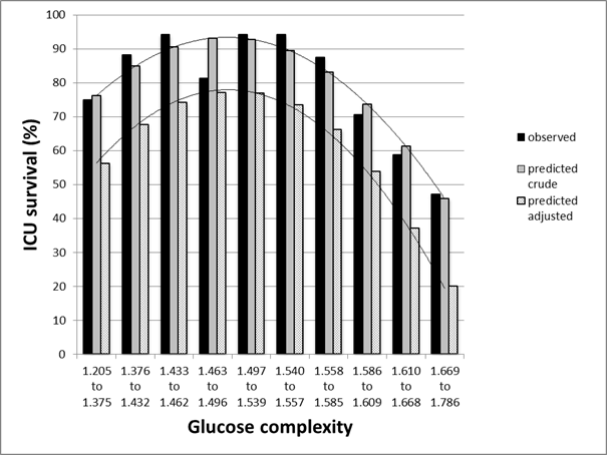
**Relation between glucose complexity and mortality**. Relation between glucose complexity and mortality can be described best with a quadratic function showing a pronounced increase in mortality with higher DFA and a moderate increase in mortality with very low DFA.

Although glucose complexity was significantly lower in non-survivors, relation between glucose complexity and mortality was not linear but can be described best with a quadratic function: logodds (ICU survival) = (-0.09 * DFA decile)^2 ^+ 0.64 * DFA decile + 1.07, where DFA decile (from 0 to 9) represents one tenth of DFA values in increasing sequence (Figure 1).

The difference of glucose complexity between survivors and non-survivors was confirmed in a binary logistic regression analysis with ICU mortality as outcome and glucose complexity, as well as age, BMI, gender, diabetes status and SAPS II as co-factors. In this model only DFA was significantly associated with mortality.

The glycemic variability measures SD, CV and GLI were significantly higher while mean daily delta glucose was significantly lower when calculated from BGA compared to CGMS values (Table 4).

**Table 4:** Impact of the method of glucose determination on variability measures.

Variability measures derived from:	CGMS values (174 patients)	BGA values (174 patients)	*P *value
Number of glucose measurements	140 209	3497	
GLI	178 ± 188	301 ± 380	<0.01
Mean daily delta (mmol/L)	4.65 ± 2.06	3.10 ± 1.50	<0.01
SD (mmol/L)	1.24 ± 0.52	1.35 ± 0.57	<0.01

BGA, blood gas analyzer; CV, coefficient of variation; GLI, glucose lability index: SD, standard deviation.

## Discussion

In this *post-hoc *analysis of CGM data the use of a real time CGM did not have an impact on measures of glycemic variability, glucose complexity and maximum glucose. The loss of glucose complexity was found to be independently associated with mortality and with the presence of diabetes mellitus.

### Glucose variability

Glycemic variability describes fluctuations of blood glucose over time and is influenced by endogenous and exogenous factors. It is associated with mortality and strategies are being sought for reducing glycemic variability. Improved glucose variability with real time CGM was reported in diabetic patients [25]. A possible explanation of real time CGM not reducing glycemic variability in the present study may be the use of an already well-established insulin titration algorithm in the control group. This algorithm has, in combination with the experienced nurses and frequent BGA measurements, already shown excellent results regarding glucose control. Hence, the use of real time CGM may have a larger benefit in environments with less experienced and established ICU staff.

Unlike numerous reports in the literature [3,4,8-10,26], we did not find a significant association between mortality and glycemic variability or between hypoglycemia and mortality, because our analysis was not powered for these purposes.

The method and frequency of glucose determination has a significant impact on variability measures as already shown with mean absolute glucose change per hour [27]. Mean daily delta was naturally higher when calculated from CGMS compared to BGA values as the gap between minimum and maximum glucose increases with the number of measured values. The increase in SD, CV and GLI calculated from BGA values may be based on the fact that blood gases are taken more frequently when a patient's glucose levels are unstable, resulting in virtually higher glucose variability values. However, glucose variability measures were calculated from CGM values in both groups in our study. Therefore, measures between these groups are comparable.

### Glucose complexity

Glucose complexity has been hypothesized as descriptive of the endogenous glucoregulatory process and is an independent predictor of mortality in critically ill patients as reported by Lundelin *et al*. for the first time [19]. These findings have now been confirmed in a larger patient group in a medical ICU. Loss of complexity in glucose time series was significantly associated with higher mortality. However, the relation between glucose complexity and mortality was not linear but can be described best with a quadratic function with a pronounced increase in mortality with higher DFA and a moderate increase in mortality with very low DFA.

The underlying hypothesis is that the ability of a healthy organism to detect even minor changes in glucose concentration and then to follow promptly with counter regulatory measures leads to a complex glucose profile. In contrast, an impaired regulatory system responds slowly and imprecisely to varying glucose concentrations and, therefore, displays low glucose complexity [19]. The unexpected mortality increase with very complex profiles, which has not been discussed by Lundelin *et al*. [19], cannot be explained by the present data and needs further investigation. Therefore, the biological explanation of the association between glucose complexity and mortality in critically ill patients should still be seen as a hypothesis.

Glucose complexity, but not SAPS II score, was significantly associated with mortality in a binary logistic regression analysis. However, this study was not powered to address this association.

Moreover, the complexity of the glycemic profile was significantly lower in diabetic, compared to non-diabetic, critically ill patients. This is consistent with several studies assessing glucose complexity in non-critically ill diabetic patients [28,29] and in critically ill patients after controlling for mortality [19]. Glucose complexity was similar between the real time CGM and the control group. We expected this finding, as glucose complexity reflects the endogenous fundamental glucose regulation, which seems autonomous from exogenous stimuli such as insulin treatment.

### Strengths and weaknesses

The present findings may be influenced by the accuracy and method of glucose monitoring. Glucose variability and inaccuracy of glucose monitoring may be positively correlated [30] and glycemic variability may be underestimated with a higher time span between glucose measurements [31].

We regard the estimation of glycemic variability in our study acceptable based on the following factors: the CGMS is relatively accurate, the accuracy of the CGMS is constant at all glucose levels [18] and the time span between glucose measurements is very small (5 minutes).

Calculation of glucose complexity is possible only with CGM. The CGMS we used in the trial was the most accurate system available at that time.

Furthermore, glucose variability and complexity measures are calculated from CGMS values in all patients. Thus, a systematic error based on the CGMS would influence both groups in the same way.

In our opinion, the advantages of the real time CGM could not be fully utilized based on the following factors. In our algorithm decisions are primarily based on the value of blood glucose, but not on the actual glucose trend [21]. However, glucose trend data is, in our opinion, one of the essential strengths of CGM. Therefore, we hypothesize that a (computer-based) algorithm using trend data for its decision process and capable of processing the great number of glucose values of CGM would be superior to the conventional algorithm used in our trials.

Moreover, the CGM devices in the study were used off-label and were originally designed for outpatients. Therefore, the display was very small and trend data could not be visualized. Due to the study design and because of the impossibility of making alarms adequately audible in an ICU environment alarm functions were not used. Devices compensating for these shortcomings are currently under development by several manufacturers, but were not available when we conducted our trial.

Consequently, despite the availability of real time data, the frequency of changes of the insulin infusion did not increase in the CGM group (Table 2). Furthermore, the number of BGA measurements was equal in both groups. However, BGA are not only used for glucose measurement in our ICU. Based on these data we conclude that the use of our CGMS device did not have a significant impact on the behavior of the nursing staff in the real time CGM group compared to the control group.

Unlike in the study of Lundelin *et al*. [19], glucose complexity metrics were convincing in our analysis based on a relatively large patient group, standardized beginning of CGM and calibration of CGM devices with glucose values determined by very accurate blood gas analyzers [32].

## Conclusions

IIT guided by real time CGM did not result in significantly reduced glycemic variability. The loss of glucose complexity was significantly associated with mortality and with the presence of diabetes mellitus. Thus, glucose complexity is an excellent measure of the endogenous glucose regulation and a robust parameter of the severity of disease in critically ill patients. In the future - when continuous glucose monitoring may become standard in the ICU - glucose complexity may add to clinical scores in this regard.

## Key messages

• IIT guided by real time CGM did not lead to reduced glycemic variability

• The loss of glucose complexity is associated with mortality and diabetes mellitus in critically ill patients

## Abbreviations

BGA: blood gas analyzer; CGM: continuous glucose monitoring; CGMS: continuous glucose monitoring system; CV: coefficient of variation; DFA: detrended fluctuation analysis; GLI: glucose lability index; Glu_SD_: overall glucose variability measured by SD; IIT: Intensive insulin therapy.

## Competing interests

The authors declare that they have no competing interests. All authors were paid by the Medical University of Vienna.

## Authors' contributions

RB collected data, carried out the statistical analyses and interpretation, and drafted the manuscript. GA collected data and revised the manuscript critically. HH performed statistical analyses. CM and UH designed and coordinated the study, collected data and helped to draft the manuscript. All authors read and approved the final manuscript.
